# Supplementary material for: Enabling preprint discovery, evaluation, and analysis with Europe PMC
Source: PLoS One. 2024 Sep 26;19(9):e0303005. doi: 10.1371/journal.pone.0303005 (PMC11426508; doi:10.1371/journal.pone.0303005)
Supplement: Attachment — Submitted filename: Response to Reviewers.docx [file pone.0303005.s001.docx]

Response to Reviewers

The authors of this manuscript sincerely thank reviewers and the editor for their valuable time and effort. Your constructive notes have helped us improve this publication. We appreciate review of the code and data along with the manuscript itself.

We have carefully considered each comment and have made every effort to address them thoroughly. Below, we provide point-by-point responses to the reviewers' comments.

We would also like to bring to your attention the change to the manuscript text regarding license restrictions for XML version of preprints posted to bioRxiv and medRxiv. The updated text within the ‘Technical challenges of indexing preprints’ section clarifies that the reuse of the XML version is governed by the license selected by the preprint authors. This change is brought on in response to preprint feedback that we have received and is also reflected in the second version of the preprint.

# Response to Reviewer 1

Lines 71-76: It was unclear from the description and analysis code whether the total number of articles in PubMed includes preprints or not. If it does, then it is appropriate to refer to the share and percentage of preprints. If not, then the measure is preprints per article and not a percentage, nor a share. Please clarify if the number of preprints is included in the total. Incidentally, when I ran the code, the result was exactly 12 and not over 12, but this is probably due to updates in the database.

***Response****: Thank you for pointing this out. Indeed, in the submitted manuscript the number of articles did not include preprints and the graph was displaying the number of preprints per article. We have changed the underlying code to calculate preprint percentage by dividing the number of preprints in Europe PMC posted in a given year by the sum of preprints and PubMed journal articles published in the same period. This change is reflected in the figure, figure legend, and in the manuscript text (line 72).*

*Regarding your note on percentage changes when running the code - this is indeed due to database updates.*

Figure 2 and lines 90-91: The numbers were higher than 30 only for the first two months, so it would be more precise to replace “few” with “two”. Using the code provided on zenodo, it seems that between 2 and 6% of the journal articles were associated with a preprint (hasPreprint == TRUE). It would be nice to reflect this in this figure, by splitting the articles into articles associated with a preprint or not. This way the conversion of preprints to journal articles becomes more apparent and complements the analysis in Fig. 6.

***Response****: We have changed “few” to “two” in the sentence on line 90.*

*Thank you for your suggestion to depict the number of journal articles based on a preprint in Figure 2. We have added a new panel to the figure that indicates the share of COVID-19 journal articles that were associated with a preprint.*

Line 112: From a security and privacy standpoint it is best to avoid using link shorteners, as they obscure the domain of the landing URL. Please replace with the original URL instead.

***Response****: Replaced with original URL as suggested.*

Lines 206-210: For better methods reproducibility it would be good to know which tools and external vendors are used for the conversion of PDF to JATS XML and to HTML.

***Response****: Thank you for your suggestion. We named the external vendor used for XML conversion and added a link to the open source XSL stylesheet used in-house to create the HTML version of the preprint.*

Lines 230-231: From the limitations given in the discussion, it seems like this method is missing cases where the title is changed from the preprint to the accepted journal version, e.g. due to reviewer requests. Please add a sentence here whether it is possible, and if yes, how to mitigate for such cases.

***Response****: Indeed existing preprint-article links can be missed by our method due to title changes. We have edited the text within the ‘‘Results’ section to clarify the limitation of the current matching algorithm, specifically related to title and first author changes (lines 413-415). We have also added a paragraph to the ‘Enriching preprint metadata’ subsection of the Discussion section to describe our plans to utilise a forthcoming automated matching mechanism developed by Crossref, which would improve matching coverage (lines 783-792).*

Table 3: The entry for BioHackrXiv is inconsistent with the other (osf) preprints. Presumably the entry for the Versioning type should be “Single identifier for all versions”. Please, double-check and correct if necessary.

***Response****: Thank you for pointing this out. We have corrected the entry for BioHackrXiv in Table 3 to “Single identifier for all versions”.*

Lines 257-258: Unless there are legal reasons to completely remove entries from the database, it would be preferable to retain the original data, for the benefit of meta-research, e.g. into research misconduct. Are the reasons for removal taken into consideration? Perhaps one or two clarifying sentences would be useful here.

***Response****: Thank you for your question. Europe PMC only removes records for preprints that have been completely erased from the corresponding server. We are not aware of the reason for deletion unless the author discloses this when contacting the Europe PMC team. We have added a clarification to the ‘Tracking changes to preprints’ subsection of the Results section (lines 450-455).*

# Response to Reviewer 2

I'm suggesting the authors consider referring to ‘preprint metasearch’ (<https://en.wikipedia.org/wiki/Metasearch_engine>), a concept that was popular in the years 1995-2005.

***Response****: Thank you for your suggestion. We have referred to Europe PMC as a metasearch solution for preprints on the line 323.*

Rationale behind the naming. Line 123 reads: “Europe PMC (https://europepmc.org/) is an open science platform and a life science literature database [35]. It is supported by a group of 35 international science funders as their repository of choice. Europe PMC is developed by the European Bioinformatics Institute (EMBL-EBI). It is part of the PubMed Central (PMC) International archive network, built in collaboration with the PMC archive in the USA. Europe”
Could the authors explain why Europe appears in the name? I understand there's a connection to the developer (EMBL-EBI) ... but is there a particular focus on the indexing of European research?

***Response****: Thank you for your question. Europe PMC is a global repository and the content it hosts is not restricted to European research.*

*The rationale behind the naming is related to the deposition services that Europe PMC provides to authors supported by its funders. Europe PMC was originally launched as UKPMC - a dedicated archive for full text open access publications supported by UK-based biomedical research funders. In 2012 the database name was changed to Europe PMC due to expansion of its funding consortium and inclusion of funding organisations based in Europe, such as the ERC, FWF, and Telethon Italy. Currently Europe PMC is supported by a group of 35 international science funders, including some that are not based in Europe or UK (such as WHO, India Alliance, and EDCTP).*

*To clarify this we have made changes to the text in the ‘Introduction’ section (lines 129-136).*

Preprint-Publication Linking (line 125). Preprint-article matching is not a trivial process. The authors sketch up how their process work. Could they be a bit more specific and compare to other research addressing this information linking task ([Cabanac et al., 2021](https://doi.org/10.1007/s11192-021-03900-7); [Eckmann & Bandrowki, 2023](https://doi.org/10.1371/journal.pone.0281659))? These 2 papers report how they perform on benchmarks, how does Europe PMC compare?

***Response****: We have added further details of the logic behind the matching process to the ‘Linking preprints to published journal articles’ subsection of the ‘Methods’ section (lines 241-246). We have described our plans to utilise a forthcoming Crossref matching mechanism, as well as plans to expand the links to include journal articles not indexed in PubMed in the ‘Enriching preprint metadata’ subsection of the Discussion section (lines 783-792).*

*Originally, our preprint-publication matching algorithm was developed back in 2018. At that time no similar algorithms were openly available for life science preprints to our knowledge to benchmark against.*

*From the beginning we have set a very high threshold for preprint-publication matching accuracy. As an archive of scholarly information it is of utmost importance that the information we share is trustworthy. Crossref metadata supplied by preprint servers and journal publishers is the primary source for preprint-publication links, and our matching mechanism has been designed to supplement that. Therefore we do not feel that benchmarking our existing algorithm against those published more recently will be beneficial, as it has been intentionally designed to be very conservative with impact on sensitivity.*

Post-Publication Peer Review: PubPeer
Table 4 lists preprint review platforms. I believe PubPeer is missing (PubPeer hosts comments on preprints, too). See for instance <https://pubpeer.com/search?q=SSRN>

***Response****: Thank you for pointing this out. It is correct that PubPeer is not included in Table 4. Preprint review information is retrieved from Crossref, Sciety and Early Evidence Base. Metadata for PubPeer reviews is not shared via these services and therefore is not available in Europe PMC. As Table 4 only lists preprint review groups with review information available in Europe PMC, we have not added PubPeer to the list.*

Success stories. The authors thought about commenting some ‘success stories’ that Europe PMC has supported (lines 631 to 652). This is useful and should be stressed with a few more examples. Which community does Europe PMC serves, why no other option could replace the Europe PMC corpus? Why is this infrastructure critically important in some research areas?

***Response****: Thank you for your suggestion as well as your questions. Throughout the publication we highlight different ways in which data and services provided by Europe PMC are used. Importance of Europe PMC as an open infrastructure for the preprint community is explained in the Introduction, within the ‘Facilitating preprint discovery with Europe PMC’ section. Europe PMC offers a free and open metasearch solution for life science preprints across many different servers for both website and programmatic users. It provides sustainable and transparent community-governed infrastructure, focussed on addressing user needs.*

*As an open life science resource Europe PMC supports many different user communities, from academic researchers, to clinicians, librarians, developers, text-miners, curators and members of the general public (Davé A, Seth V, Pottinger E, Rosenberg C, Potau X, Varnai P. Measuring the Value and Impact of the Europe PMC Repository [Internet]. Wellcome Trust; 2019. Available from: https://wellcome.figshare.com/articles/online_resource/Measuring_the_Value_and_Impact_of_the_Europe_PMC_Repository/8326559/2).*

*We highlight Europe PMC’s importance for the systematic review community in ‘Supporting preprints inclusion in meta-analyses and systematic reviews’ section within Discussion and reference multiple studies that have used Europe PMC preprint search for evidence synthesis. We describe the impact of Europe PMC services for text-mining and programmatic analyses in the ‘Providing programmatic access for preprints’ section and reference several use cases.*

*To incorporate your suggestion, we have expanded the latter section to include another use case of Europe PMC API from Sciety, a platform for reviewing and curating preprints in the life sciences (lines 665-666). We have also described our outreach efforts to increase the number of preprints with license information in Crossref, supporting programmatic preprint reuse and their inclusion by other discovery services (lines 677-687).*

*As an open scholarly infrastructure Europe PMC does not impose any barriers to access its APIs and bulk downloads. Users do not need to register or sign in to retrieve data through these services. This makes it difficult to track Europe PMC use. Use cases cited in this publication were brought to our attention either through personal communication or through literature search for publications citing use of Europe PMC.*

*While we are aware of other use cases, including Zenodo, Dimensions, or OpenCitations (https://europepmc.org/API-case-studies), they are not specific to preprints. Therefore we chose to omit them when writing this article.*

Non Crossref DOIs. Line 666 reads: ‘some do not’. Could the author try and quantify ‘some’. I suppose that's a minority ... and this should be clarified to understand what's missing in terms of coverage here. I saw the list of repositories on line 733 but failed to grasp the ‘magnitude of the loss’ (percentage of all preprints, say).

***Response****: Thank you for your question. We are not aware of any continuously updated community resource that tracks life science preprints servers. Without a complete list of relevant servers we cannot quantify how many of them do not register Crossref DOIs.*

*To ensure better coverage, we continually check for new servers that should be added to Europe PMC using community resources, such as preprint archives content coverage in search services (*[Scholarly search engine comparison](https://docs.google.com/spreadsheets/d/1ZiCUuKNse8dwHRFAyhFsZsl6kG0Fkgaj5gttdwdVZEM/edit?gid=1560823935#gid=1560823935)*) and list of preprint servers from ASAPbio (*[*https://asapbio.org/preprint-servers*](https://asapbio.org/preprint-servers)*). If a server does not register Crossref DOIs, but fulfils other inclusion criteria, including free API access, we would consider including it in Europe PMC.*

*To clarify this in our publication we have made text changes to the corresponding paragraph and added examples of preprint servers that contain some life science preprints and do not register Crossref DOIs (lines 699-704).*

In Table 1, add an estimate of the number of preprints hosted at each server

***Response****: Thank you for your suggestion. The number of preprints for each server included in Europe PMC as of 15 September 2023 is available in our previous publication (Rosonovski S, Levchenko M, Bhatnagar R, et al. Europe PMC in 2023. Nucleic Acids Research. 2024 Jan;52(D1):D1668-D1676). As the number of preprints in the database changes daily, we provide the search syntax for preprints from each server in Table 1. Readers can use the search syntax to get an accurate number of preprints at any point in the future.*

line 257 reads ‘and not even a notification remains.’ Indeed, this has been documented for SSRN for instance: <https://retractionwatch.com/2021/05/17/preprints-are-works-in-progress-the-tale-of-a-disappearing-covid-19-paper/>

***Response****: Thank you for bringing this to our attention.*

Biological annotations (l290): How about RRIDs? <https://scicrunch.org/resources>

***Response****: RRID mentions in preprints are routinely identified using text-mining and are available as a sub-type of accession number annotations. See results for* [*(ACCESSION_TYPE:rrid) AND (SRC:PPR)*](https://europepmc.org/search?query=%28ACCESSION_TYPE%3Arrid%29%20AND%20%28SRC%3APPR%29&page=1) *search query that brings up preprints mentioning RRIDs.*

line 335: Which search engine technology runs Europe PMC? (e.g., Lucene, Solr, Elasticsearch?)

***Response****: We have added a sentence to indicate that Europe PMC uses the Solr platform as the underlying search engine technology (lines 350-351).*

line 395: on publications following a preprint, one should perhaps acknowledge that some preprints will stay as such and no journal article will follow (because the authors decide not to).

***Response****: We have added a sentence to the beginning of the paragraph to explain that some preprints are intended as the final version of record (lines 401-402).*

line 1028: extra space to be removed: ‘Covid- 19’

***Response****: Thank you for pointing this out. At present there is no extra space in the term ‘Covid-19’ on the line 1028.*

Figure 5: add the percentage above each bar.

***Response****: Thank you for your suggestion. We have added a percentage for preprints with more than one version to the text (line 394). We did not add percentages to the bars in Figure 5, as the graph only displays the number of preprint records that have more than one version in Europe PMC and therefore excludes preprints with only 1 version which comprise 90% of the total.*

# Response to the Editor

Please make sure that you indicate whether your protocol was pre-registered or not. If not, please give an explanation and add some words in the limitation section.

***Response****: Our study does not include a pre-registered protocol. At the time when we started indexing life science preprints it was still a newly emerging concept. We needed to adopt a flexible and agile approach that would allow us to incorporate community feedback and respond to changes quickly. Instead of publishing a pre-registered protocol we have continuously shared our plans and progress in a public roadmap.*

Please add a few words about limitations in the abstract and add a specific "limitations" section that summarize the main limitations of your work. This is in order to avoid any spin.

***Response****: This publication addresses two types of limitations - those arising from the technical approach taken by Europe PMC and those imposed by variability in practices and metadata standards that exist between preprint platforms. The latter are described in the ‘Technical challenges of indexing preprints’ section within Discussion. To address your point we have now renamed this section to ‘Limitations and technical challenges of indexing preprints’.*

*The limitations of our indexing approach are described throughout the text within relevant sections. For example, the Methods section on ‘Handling preprint versions’ describes the limitations of recording version history for preprints that share DOIs, and this is expanded on in the ‘Tracking preprint changes’ section within Results. Discussing limitations in relevant sections allows us to maintain the necessary context and help readers understand the nuances of the preprint indexing process.*

*While we believe that embedding the limitations within the main text serves our readers more effectively in the case of this particular publication, we are open to further discussion and would appreciate your guidance on the matter.*

Please select the most appropriate guideline from the equator network.

***Response****: The EQUATOR Network provides a list of reporting guidelines for health research literature. While Europe PMC provides tools that can be used for health research, results described in this publication do not directly relate to human health outcomes. This publication can be considered methodological research, which is out of scope for EQUATOR Network guidelines. We have therefore not been able to find a suitable guideline.*
